# ContactPFP: Protein Function Prediction Using Predicted Contact Information

**DOI:** 10.3389/fbinf.2022.896295

**Published:** 2022-06-02

**Authors:** Yuki Kagaya, Sean T. Flannery, Aashish Jain, Daisuke Kihara

**Affiliations:** ^1^ Department of Biological Sciences, Purdue University, West Lafayette, IN, United States; ^2^ Department of Computer Science, Purdue University, West Lafayette, IN, United States

**Keywords:** function prediction, residue contact prediction, gene function, functional genomics, protein structure, PFP

## Abstract

Computational function prediction is one of the most important problems in bioinformatics as elucidating the function of genes is a central task in molecular biology and genomics. Most of the existing function prediction methods use protein sequences as the primary source of input information because the sequence is the most available information for query proteins. There are attempts to consider other attributes of query proteins. Among these attributes, the three-dimensional (3D) structure of proteins is known to be very useful in identifying the evolutionary relationship of proteins, from which functional similarity can be inferred. Here, we report a novel protein function prediction method, ContactPFP, which uses predicted residue-residue contact maps as input structural features of query proteins. Although 3D structure information is known to be useful, it has not been routinely used in function prediction because the 3D structure is not experimentally determined for many proteins. In ContactPFP, we overcome this limitation by using residue-residue contact prediction, which has become increasingly accurate due to rapid development in the protein structure prediction field. ContactPFP takes a query protein sequence as input and uses predicted residue-residue contact as a proxy for the 3D protein structure. To characterize how predicted contacts contribute to function prediction accuracy, we compared the performance of ContactPFP with several well-established sequence-based function prediction methods. The comparative study revealed the advantages and weaknesses of ContactPFP compared to contemporary sequence-based methods. There were many cases where it showed higher prediction accuracy. We examined factors that affected the accuracy of ContactPFP using several illustrative cases that highlight the strength of our method.

## 1 Introduction

Proteins are working molecules in a cell. Virtually all cellular functions are carried out mainly by proteins. Therefore, elucidating the biological function of proteins is a central problem in molecular biology, biochemistry, genetics, and genomics. Ultimately, the function of proteins needs to be determined by experiments. However, in the process of experimental elucidation of protein function, computational function prediction is very useful for guiding experiments by, for example, helping biologists construct hypotheses in designing experiments.

As sequencing the whole genome has become a standard experimental protocol for studying an organism, many protein sequences are now available in various databases (Sayers et al., 2021), and many of them remain unannotated. Thus, there is an increasing need for computational function prediction. Indeed, computational function prediction has been one of the most extensively studied topics in bioinformatics (Hawkins and Kihara, 2007). Conventionally, protein function annotation has been performed through sequence similarity search tools, which use BLAST (Altschul et al., 1990) or FASTA (Lipman and Pearson, 1985), and motif searches (Bairoch and Bucher, 1994; Mistry et al., 2021). In addition to such sequence-based methods (Hawkins et al., 2006; Chitale et al., 2009; Jain and Kihara, 2019), other approaches have been explored, which use omics-data (Obayashi et al., 2019; Szklarczyk et al., 2019), phylogenetic profiles (Pellegrini et al., 1999), and 3D structures of proteins (Sael and Kihara, 2010; Sael and Kihara, 2012; Zhu et al., 2015). As also observed in recent community-wide assessments for computational function prediction, the Critical Assessment of Function Annotation (CAFA), methods that combine different information sources by machine learning often showed relatively strong prediction performance (Khan et al., 2019; You et al., 2019).

In this work, we used protein 3D structure information for inferring the function of proteins. It has been long known that the 3D structures are better conserved than protein sequences during evolution (Chothia and Lesk, 1986), and thus they help capture distant functional relationships of proteins (Das et al., 2021). However, the 3D structure information has not been much used in practice in function prediction because the 3D structure has not been determined experimentally for many proteins. However, the situation has been changing due to recent progress in the protein structure prediction field, which has made significant improvements in amino acid residue contact and distance map prediction (Greener et al., 2019; Xu, 2019; Jain et al., 2021; Maddhuri Venkata Subramaniya et al., 2021). It may be noted that the accuracy of models by Alphafold (Jumper et al., 2021), the top-ranked structure prediction method in the recent Critical Assessment of techniques in protein Structure Prediction (CASP) (Abriata et al., 2019), often reach the level of experiments, such as X-ray crystallography. By using such a recent protein structure prediction method, it is now possible to compensate for the limited availability of the structural information of proteins. Thus, we now have an unprecedented opportunity for structure-based functional inference for nearly all proteins in genomes since their amino acid sequences are available, even if their 3D structures are not.

Here, we explore how protein structure information, particularly amino acid contact information, can contribute to the accuracy of function prediction. To do so, we developed a new protein function prediction method, ContactPFP. In ContactPFP, instead of performing sequence-based database search, a query protein is compared with proteins in a database in terms of predicted contact maps. Since an amino acid contact map is, in principle, sufficient to build a 3D structure of the protein, using contact maps is conceptually equivalent to considering 3D structure similarity. We benchmarked ContactPFP on a dataset of 9,642 proteins and compared its performance with sequence-based function prediction methods that performed among the top in CAFA. The benchmark revealed the strengths and weaknesses of ContactPFP. We report the performance of ContactPFP relative to several key parameters. Also, to characterize ContactPFP’s performance, we discuss examples where ContactPFP showed its strengths and cases where ContactPFP did not perform as well as the sequence-based methods that were compared against.

## 2 Materials and Methods

### 2.1 Overview of the ContactPFP Method

**FIGURE 1:**
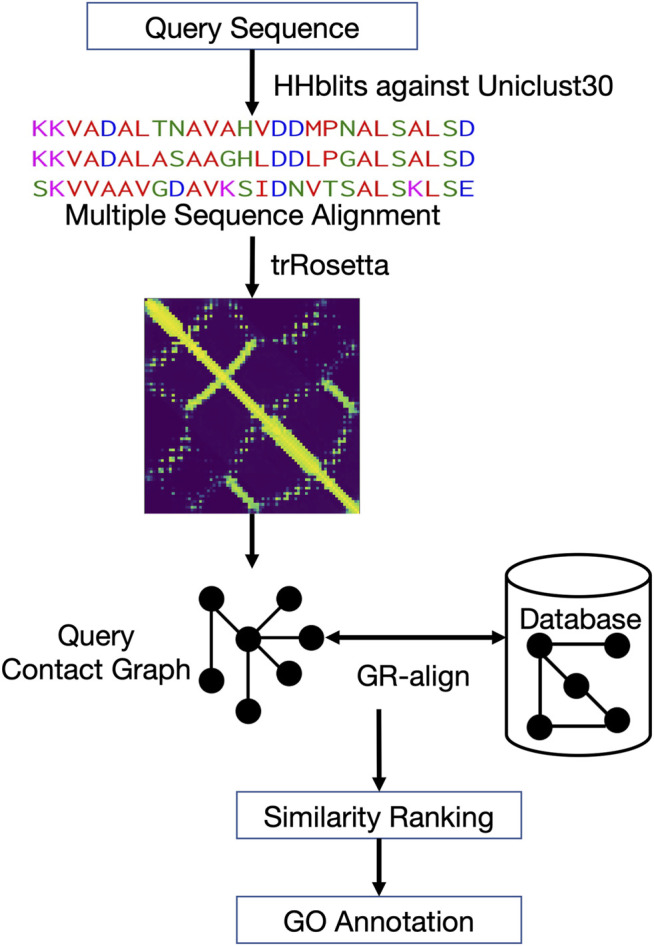
Overview of ContactPFP. From an input protein sequence, residue-residue contact information is predicted with trRosetta, which is represented as a graph. Then, the graph is compared with contact map graphs in a database using GR-Align. Proteins in the database are sorted by graph similarity to the query and GO terms are extracted from top hits.

Figure 1 shows the workflow of ContactPFP. For a query protein sequence, ContactPFP constructs a multiple sequence alignment (MSA) using HHblits (Steinegger et al., 2019), that is, run against the Uniclust30 database (Mirdita et al., 2017) with a parameter set of “-n 3 -id 99 -cov 50 -diff inf”. Then, using the MSA, residue-residue distance prediction (a distance map) is computed for a query using trRosetta (Yang et al., 2020). The predicted distance map of the query is then compared with contact maps of proteins in the reference database using the GR-Align algorithm (Malod-Dognin and Prẑulj, 2014). Since GR-Align compares contact maps of proteins, predicted distance maps were converted to contact maps, or contact graphs, where nodes represent amino acid residues and edges connect residue pairs that are closer than a distance cutoff value. As the distance cutoff values to define a contact, we used 8, 10, and 12 Å between between Cβ atoms. For a given contact distance cutoff (e.g., 8 Å), we define a residue pair as “in contact” if the probability that the pair has the cutoff distance or closer between each other is 0.5 or larger.

The reference database was constructed from Swiss-Prot (The UniProt Consortium, 2021). Sequences shorter than 20 residues and longer than 2000 residues, which made up 1.1% of the all sequences, were excluded. For the remaining 555,378 proteins (98.9%), contact graphs were computed as described above. Using GR-Align, the query contact graph is compared with all contact graphs in the reference database, which are then ranked by graph similarity to the query. In GR-Align, two contact graphs are aligned so that the similarity score of the graphs, which considers graphlet distribution similarity of mapped nodes, is maximized. This graphlet degree similarity can capture the local similarity of contact graphs. A graph similarity score by GR-Align ranges from 0 to 1.0, with 1.0 indicating an exact match in graphlet distributions. Proteins in the reference database that have a similarity score of 0.5 or higher by GR-Align were considered as hits. GO terms from hits were collected and weighted by the sum of the graph similarity scores of hits that have the GO terms. The score of a predicted GO term *i* is computed as follows:

$$GOScore\left(i\right)=\;\underset{k\in Protein\;hits\;with\;GO\left(i\right)}{\sum }Graph\_SimScore\left(k\right)$$ (Eq. 1)

where k is a hit (i.e., graph similarity score > = 0.5 to the query) that has the GO term *i* in its annotation. Finally, predicted GO terms for a query is normalized by the highest score among them, so that the most confident GO term has a score of 1.0.

### 2.2 Constructing the Function Annotation Database

GO terms for proteins in the reference database were compiled from 12 data sources. The primary database used was the UniProtKB/Swiss-Prot. GO terms with IEA (Inferred from Electronic Annotation) evidence code (Boutet et al., 2016) were also included because considering IEA gave a higher function prediction accuracy than excluding them in our previous works (Chitale et al., 2009; Hawkins et al., 2009). In addition to UniProtKB/Swiss-Prot, we integrated annotations from UniPathway (Morgat et al., 2012), TIGRFAMs (Haft et al., 2013), SMART (Letunic et al., 2021), Reactome (Jassal et al., 2020), PROSITE (Sigrist et al., 2013), ProDom (Bru et al., 2005), PRINTS (Attwood et al., 2012), PIRSF (Nikolskaya et al., 2006), Pfam (Finn et al., 2016), InterPro (Finn et al., 2017) and HAMAP (Pedruzzi et al., 2015). This database extension contributed an additional 8,727 GO terms to our reference database. We showed in our previous work that this annotation expansion has a positive effect on function prediction performance (Khan et al., 2015).

### 2.3 Constructing the Benchmark Dataset

We started from representative sequences in UniRef50 (Suzek et al., 2015) (22 October 2019 version). We filter out entries that do not fall within lengths of 100–2000. We only considered the entry names that existed in the 11 November 2019 version of Swiss-Prot. We kept only entries with at least one experimentally verified GO Term in all three categories. To remove the potential redundancy of annotations in the dataset, we only kept one protein from homologous proteins from different organisms. For example, among the two entries, ZRT1_SCHPO and ZRT1_YEAST, which were originally included in the representative sequences, we kept only ZRT1_SCHPO. The ortholog proteins were identified by the common mnemonic protein identification code, e.g.,“ZRTI”. Further, to remove the sequence redundancy, we performed sequence clustering by MMseqs2 (Steinegger and Söding, 2017) with a 25% identity and a 80% coverage (--min-seq-id 0.25 -c 0.8). As a result, we had 9,642 sequences in the benchmark dataset.

### 2.4 Existing Methods Used as Reference

To characterize the performance of ContactPFP, we compared it with four sequence-based methods, PSI-BLAST (Altschul et al., 1997), PFP (Hawkins et al., 2009), ESG (Chitale et al., 2009), and Phylo-PFP (Jain and Kihara, 2019). PSI-BLAST is considered the baseline of function prediction methods. We selected PFP, ESG, and Phylo-PFP because they are sequence-based methods that performed well in CAFA challenges (Radivojac et al., 2013; Jiang et al., 2016; Zhou et al., 2019). Our group, who used these three methods, were among the best teams in the series of CAFA challenges. PFP, ESG, and Phylo-PFP use PSI-BLAST search results in different elaborate ways: In PFP, GO terms extracted from PSI-BLAST hits are scored by the sum of the negative logarithm of E-value of the hits. Thus, GO terms from hits with smaller E-values are scored higher. Up to 20,000 hits were considered. In ESG, the top hits of the first PSI-BLAST run are used to perform a second round of database search. In Phylo-PFP, retrieved sequences were ranked by considering both raw E-value and the edge distance on a phylogenetic tree constructed for the sequence. In ESG and Phylo-PFP, raw scores of predicted GO terms are normalized to a range between 0 and 1.0 by the highest score observed for the target protein.

These sequence-based methods identify the query itself as the top hit in a database search. To avoid taking GO terms from the query itself for PFP, ESG, and Phylo-PFP, we removed the query and all hits that had an E-value of 0.0 in the last round of PSI-BLAST before extracting GO terms. These excluded proteins were also removed from the hit list of ContactPFP. For PSI-BLAST, we extracted GO terms from the top 10 hits (except for the query itself and hits with 0 E-value) in the third iteration of a PSI-BLAST run and assigned a score of 1.0 to all the predicted terms.

## 3 Results

### 3.1 Effect of the Residue-Contact Definition and the Fold Similarity

**FIGURE 2:**
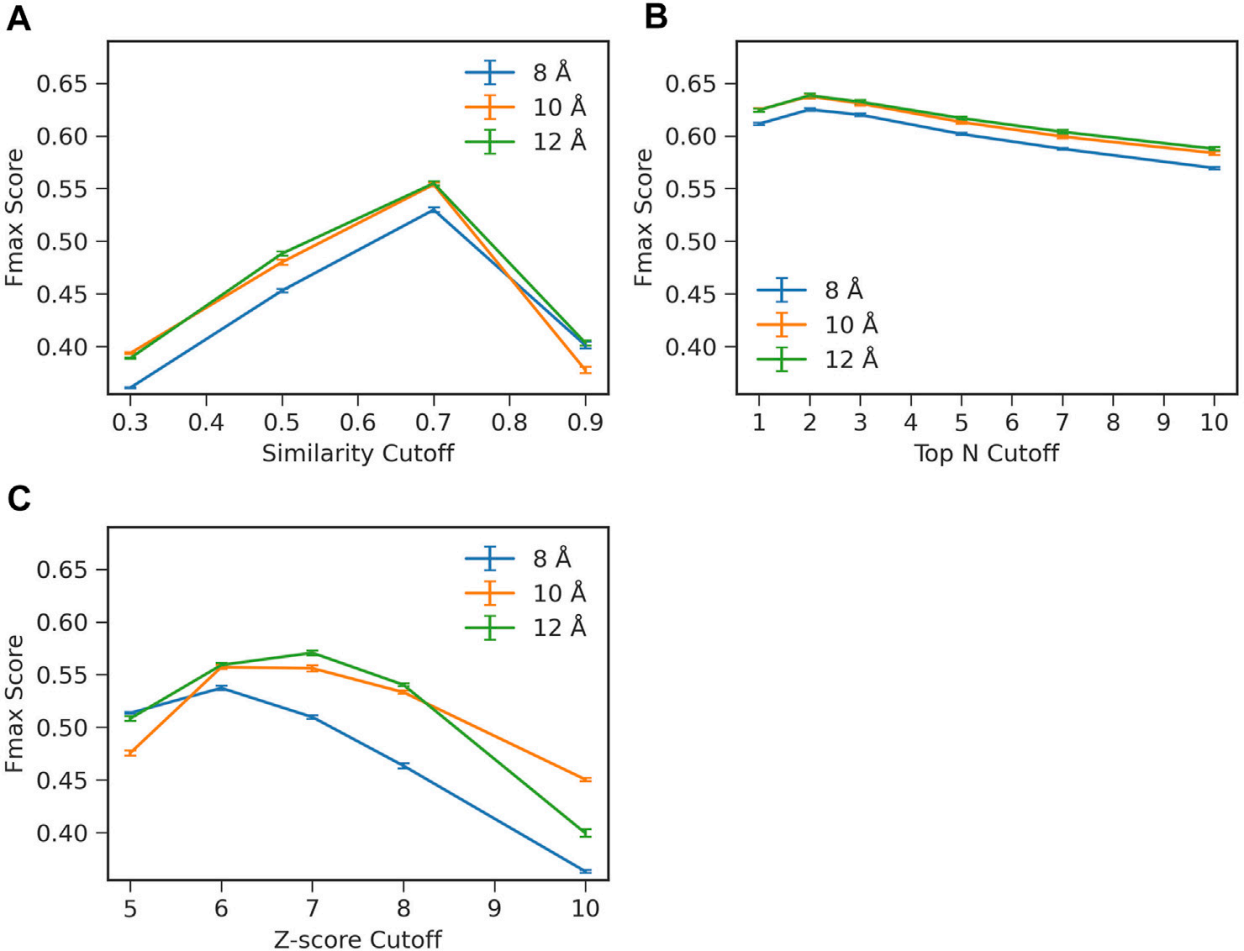
Influence of parameters on the prediction performance of ContactPFP. Parameters were examined that determine the definition of hits in the contact map database search. The y-axis shown is the average Fmax score computed for the four test sets in the four-fold cross validation. In each plot, three distance cutoffs, 8, 10, 12 Å, were used that defined residue contacts. The bar indicates the standard deviation calculated from four-fold cross validation. **(A)** Raw contact map graph comparison score. From a database search result, we only considered retrieved proteins with a specified graph similarity score or higher. The average standard deviation was 0.002. **(B)** Selecting top N hits by the raw score. In this scheme, we only selected top N hits as specified on the x-axis regardless of their scores. The average standard deviation was 0.002. **(C)** Z-score of the contact map graph comparison score. In this scheme, we chose hits to consider by the Z-score of the graph similarity score relative to the score distribution of the entire reference database. The average standard deviation was 0.002.

To start with, we examined how two important hyperparameters in ContactPFP, the definition of residue-residue contacts and choices of top hits from a database search, affect the function prediction accuracy (Figure 2). When constructing a graph from residue distance prediction, a choice of residue distance cutoff needs to be made. A larger distance connects more residue pairs making more edges in a contact graph, while a smaller cutoff would highlight densely connected domains (Yuan et al., 2012). We tested three distance cutoffs between Cβ atoms, 8, 10, and 12 Å, to define residue-residue contacts. The latter parameter, the choice of top hits, decides which proteins from the database search to use for extracting GO terms for annotating the query protein. Increasing this similarity level reduces the number of hits to consider while decreasing the cutoff leads to an increased number of hits.

To examine the effect of the parameters, we performed a four-fold cross validation. In combination with the distance cutoffs, we examined three different ways to select top hits from a search (Figure 2). The Fmax score shown in the panels in Figure 2 are the average values of the four test sets used in the cross validation. In Figure 2A, we used the raw graph similarity score from GR-Align to select hits from a database search. Retrieved proteins in a search that have a similarity score lower than a specified cutoff were discarded. Among the four scores examined, 0.3, 0.5, 0.7, and 0.9, the highest Fmax score of 0.555 was observed when a graph similarity score of 0.7 was used in combination with a distance cutoff of 12 Å. In Figure 2B, we used the top N hits from a database search to extract GO terms regardless of their graph similarity score. The highest Fmax score, 0.638, was achieved when the first two hits were used (i.e., *n* = 2) with a distance cutoff of 12 Å. In the last panel, Figure 2C, we considered the Z-score of the graph similarity score to select top hits. The highest Fmax score, 0.571, was achieved with a Z-score of 7 using a residue distance cutoff of 12 Å. In each panel in Figure 2, the standard deviations from the four-fold validation were small, 0.002, and the best parameter combinations were consistent across the four-fold.

Overall, the combination of 12 Å for the distance cutoff and using the top 2 hits showed the best performance among the conditions tested. Therefore, we report the results with this condition in the subsequent sections. Regarding the contact distance cutoff, 12 Å showed the best performance in all three panels, which is consistent with what the GR-Align paper reported (Malod-Dognin and Prẑulj, 2014).

### 3.2 GO Term Prediction Performance of ContactPFP

We now report the overall performance of ContactPFP in Table 1 in comparison to the other four methods. Two values are reported in Table 1. In terms of the average Fmax score (the left column), ContactPFP’s performance was the second-highest, slightly lower than Phylo-PFP. ESG, PFP, PSI-BLAST followed in this order. Breakdown of the performance in the three GO categories (Table 2) showed essentially the same trend. ContactPFP was the second in Cellular Component, the third in Molecular Function, and the second in the Biological Process.

**TABLE 1:** The average Fmax score of ContactPFP and the other four methods on the benchmark dataset.

Method	Fmax	Wins by ContactPFP
ContactPFP	0.638	-
Phylo-PFP	0.662	5,357 (55.6%)
ESG	0.634	5,452 (56.5%)
PFP	0.586	5,940 (61.6%)
PSI-BLAST	0.574	6,386 (66.2%)

The count of benchmark proteins in which ContactPFP performed better than the other methods is shown in the third column. For ContactPFP, the top 2 hits from a search were used to extract GO terms.

**TABLE 2:** The average Fmax score and Smin score in the three GO categories.

Method	Fmax				Smin			
	ALL	CC	MF	BP	ALL	CC	MF	BP
ContactPFP	0.638	0.718	0.728	0.606	95.042	16.294	12.319	66.341
Phylo-PFP	0.662	0.75	0.759	0.641	98.985	15.067	13.376	70.48
ESG	0.634	0.714	0.746	0.598	106.368	16.541	13.227	76.673
PFP	0.586	0.698	0.689	0.562	117.773	18.055	16.365	82.991
PSI-BLAST	0.574	0.655	0.678	0.544	281.223	44.163	38.382	198.695

CC, cellular component; MF, molecular function; BP, biological process.

**FIGURE 3:**
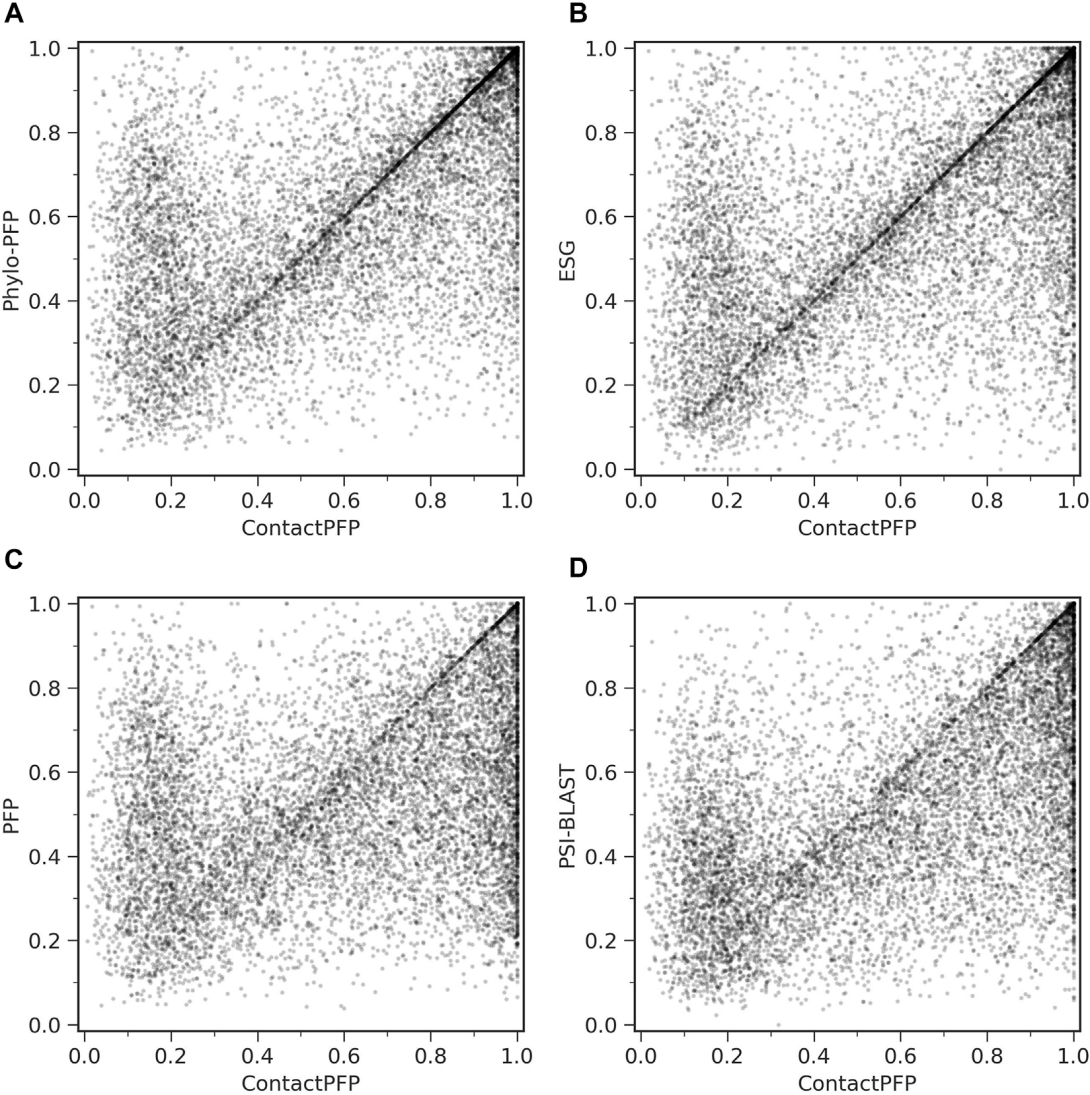
Comparison of Fmax score of individual target proteins. To be precise, they are F-score of each protein using the score cutoff that yielded the Fmax score of the benchmark dataset. Each point represents a protein in the benchmark dataset. **(A)** Comparison between ContactPFP and Phylo-PFP; **(B)** Comparison between ContactPFP and ESG; **(C)** comparison between ContactPFP and PFP; **(D)** Comparison between ContactPFP vs. PSI-BLAST.

On the other hand, when predictions given to individual target proteins were compared between two methods (Table 1, the right column), more than half of the proteins (55.6%) had predictions with a higher Fmax score by ContactPFP than Phylo-PFP. ContactPFP also had more wins over ESG, PFP, and PSI-BLAST. Thus, in this head-to-head comparison, ContactPFP was the best. To understand how the performance of the methods differ, we showed the Fmax score of individual target proteins by ContactPFP and each of the other methods in scatter plots (Figure 3). The scores seem not to distribute randomly. Rather, they show an interesting pattern of a “mirror-imaged N-shape”, where there are a substantial number of targets with around 1.0 Fmax as well as other targets with around 0.1 by ContactPFP. This distribution implies that ContactPFP may have both a characteristic strength and weakness when compared with the other methods.

In Table 2, we also presented the Smin score of the five methods. Smin evaluates remaining uncertainty/missing information from predicted GO terms (Jiang et al., 2016). The lower, the better prediction. In terms of Smin, ContactPFP is the best among the all five methods when all GO categories, MF, or BP are considered. ContactPFP was the second in the CC category following Phylo-PFP.

### 3.3 Effect of the Contact Prediction Accuracy

**FIGURE 4:**
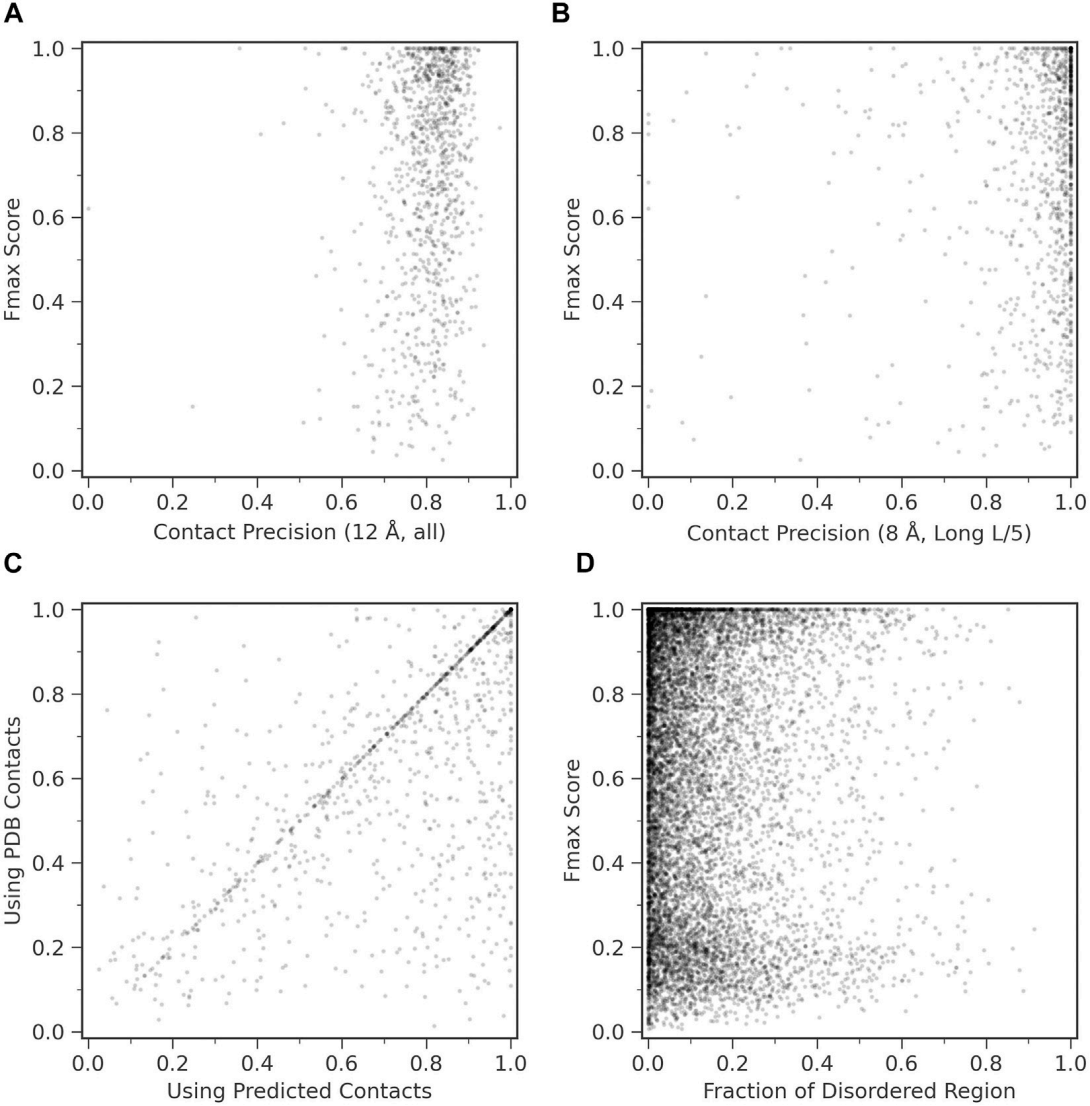
Function prediction accuracy relative to structural features of target proteins. **(A)** and **(B)**, Fmax score of ContactPFP relative to the precision of contact prediction. Each point is corresponding to a protein which has an experimentally determined structure. There were 1,029 proteins of them. **(A)** Fmax score relative to the precision of all predicted contacts. Contacts are defined for residue pairs that have a Cβ distance within 12 Å from each other. The average precision was 0.801. **(B)** Fmax score relative to the precision when we considered the top L/5 predicted long-range contacts, which were defined as contacts that are 24 residues or more apart on the sequence. L is the length of a protein. Contacts were defined as residue pairs that have their Cβ atoms placed within 8 Å. The average precision L/5 long precision was 0.908. **(C)** Comparison of the performance between ContactPFP using predicted contacts and ContactPFP that uses accurate contacts taken from the experimentally determined structures. Contacts are defined for residue pairs that have Cβ atoms within 12 Å from each other. Fmax scores of the 1,029 targets that have PDB structures were compared. **(D)** The effect of the fraction of disordered regions in proteins to the Fmax score. We used fldpnn to predict residues in disordered regions.

We examined how contact prediction accuracy affects to the GO prediction accuracy in ContactPFP. For this analysis, we used 1,029 targets in the benchmark dataset, which have an experimentally determined protein structure that covers more than 80% of the residues in the target protein. If we consider precision of all predicted contacts (Figure 4A), all the targets fall into contact precision around 0.8 (the average: 0.801), and we found no correlation between the Fmax score. The conclusion was the same when we only considered long-range contacts predicted within the top L/5 scores (Figure 4B); no correlation was observed.

In Figure 4C, we further examined what would happen if we used completely accurate contacts for targets that were taken from experimentally determined structures. Interestingly, the Fmax score was higher when predicted contacts were used. The Fmax score of ContactPFP using predicted contacts and accurate contacts were 0.744 and 0.658, respectively. This is mostly because we use the reference database of proteins with predicted contacts. Similar proteins are likely to have similar predicted contact patterns, either accurate or inaccurate, and the similarity can be captured by contact graph comparison.

### 3.4 Effect of Disordered Regions

We were also curious how ContactPFP performs for proteins that have intrinsic disordered regions (IDRs) because an IDR does not usually form residue contacts. In Figure 4D, we examined Fmax scores of target proteins relative to the fraction of IDRs in a protein. IDRs were predicted with fldpnn (Hu et al., 2021). To make disorder predictions more reliable, we used SPIDER3-single (Heffernan et al., 2018) to predict secondary structure of proteins and only considered residues which were also predicted as loops (class C) SPIDER3-single as the final disorder residues. We did not observe clear correlation between Fmax scores and the fraction of IDRs.

### 3.5 Case Studies

In this section, we discuss cases that illustrate ContactPFP’s performance relative to sequence-based methods.

#### 3.5.1 Case 1: Outer Membrane Porin G

The first example shows a successful prediction by ContactPFP for outer membrane porin G (UniProt ID: P76045). This protein is present in the outer membrane of *E. coli*, for which GO terms such as “cell outer membrane” (GO: 0009279) are annotated in the CC category. This protein is transmembrane and transports sugars from outside to inside the cell. This corresponds to “maltose transporting porin activity” (GO: 0015481). For this protein, ContactPFP showed a high prediction accuracy, a Fmax score of 0.754, while it was 0.083, 0.140, and 0.085 for PFP, ESG, and Phylo-PFP, respectively. Table 3 shows predicted correct and incorrect GO terms by the methods. ContactPFP was able to predict three out of four correct GO terms with the highest score of 1.0 and the remaining term (pore complex) with a score about half of the highest score (0.473). In contrast, Phylo-PFP, ESG, and PFP assigned low scores to the correct terms and instead selected wrong GO terms that do not exist in the UniProt entry with the highest (1.0) or with high scores over 0.9.

**TABLE 3:** Predicted GO terms for outer membrane porin G (UniProt ID: P76045).

		Correct GO terms	Confidence Score			
			ContactPFP	Phylo-PFP	ESG	PFP
MF	GO:0015481	Maltose transporting porin activity	-	-	-	-
MF	GO:0015478	oligosaccharide transporting porin activity	-	-	0.001	-
MF	GO:0015288	porin activity	1.000	0.283	0.090	0.250
BP	GO:0034219	carbohydrate transmembrane transport	-	0.432	0.053	0.490
BP	GO:0006811	ion transport	1.000	0.458	0.087	0.510
CC	GO:0009279	cell outer membrane	1.000	0.345	0.092	0.380
CC	GO:0045203	integral component of cell outer membrane	-	0.099	0.062	0.110
CC	GO:0046930	pore complex	0.473	0.099	0.090	0.110
		**Incorrect GO terms**	**ContactPFP**	**Phylo-PFP**	**ESG**	**PFP**
MF	GO:0046872	metal ion binding	-	1.000	0.272	1.000
BP	GO:0007155	cell adhesion	-	0.975	0.161	1.000
CC	GO:0005737	cytoplasm	-	1.000	0.097	0.920

All correct GO terms assigned in the UniProt entry are listed. For incorrect GO terms, GO terms that illustrate the difference between ContactPFP and the other methods are shown. Scores assigned to GO terms by the methods were normalized by the highest GO term score for this target protein. Thus, 1.0 means it is the top (i.e., most confident) prediction by the method for this protein.

**FIGURE 5:**
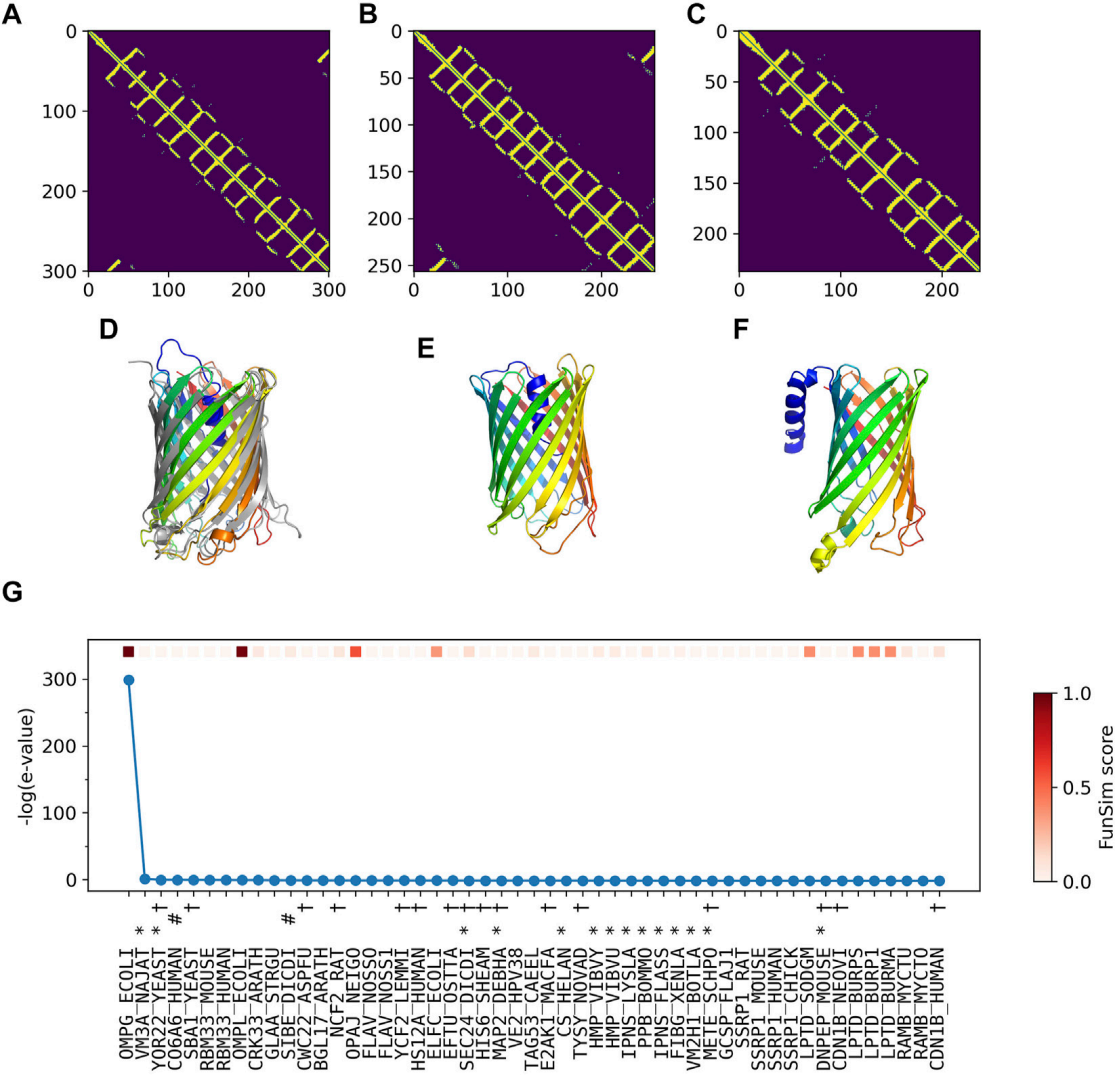
GO prediction by ContactPFP for outer membrane porin G (P76045). The first three panels **(A–C)** and the subsequent panels in the second row **(D–F)** are predicted residue contacts and resulting protein structure models. **(A)** The predicted contact maps of the query, OMPG_ECOLI (P76045). Residue pairs predicted to be in contact are shown in yellow. **(B)** The predicted contact maps of YAIO_ECOLI (Q47534), the most similar contact map with the GR-align score of 0.733 **(C)** The predicted contact maps of NANC_ECOL6 (P69856). The second closest contact map with the GR-align score of 0.658. GO terms of these two proteins were used for the prediction. **(D)** The predicted structure of OMPG_ECOLI (P76045) was generated by trRosetta (rainbow) superimposed with PDB structure 2X9K (gray). The root mean square deviation (RMSD) of the model to the native is 3.63 Å. **(E)** The predicted structure of YAIO_ECOLI (Q47534) was generated by trRosetta (rainbow). For this protein, no experimental structure has been reported. **(F)** The predicted structure of NANC_ECOL6 (P69856) was generated by trRosetta (rainbow). No experimental structure was reported for this protein. **(G)** The top hits for OMPG_ECOLI by PSI-BLAST search against Swiss-Prot. The query itself is shown in the first position. Funsim functional similarity scores (Schlicker et al., 2006; Hawkins et al., 2009). The three categories of each protein compared with the query are shown in the top row in a color scale. The y-axis shows the sequence similarity in the form of -log_10_ (E-value). The proteins that have incorrect GO terms listed in Table 3 are marked with symbols: *, “metal ion binding” (GO: 0046872); #, “cell adhesion” (GO: 0007155); and †, “cytoplasm” (GO: 0005737).

In Figure 5, we analyzed how the correct GO prediction was possible by ContactPFP. The query protein has the β barrel fold, which was well predicted by trRosetta (Figures 5A,D). With the accurate contact prediction of the query, ContactPFP was able to identify two other outer membrane proteins, YaiO (YAIO_ECOLI) and probable N-acetylneuraminic acid outer membrane channel protein NanC (NANC_ECOL6) from *E. coli* O6:H1, that also have a β barrel fold (Figures 5B–F). These two proteins have correct GO terms, GO:0009279 (cell outer membrane), GO:0015288 (porin activity), GO:0006811 (ion transport), GO:0046930 (pore complex), and GO:0008643 (carbohydrate transport), which is a parent, more-general term of a correct term, GO:0034219 (carbohydrate transmembrane transport). Since these top 2 most similar structures were used for GO term transfer, ContactPFP was able to make the correct GO predictions in Table 3. The query protein has been reported to have low sequence similarity with other porin proteins with similar functions (Subbarao and van den Berg, 2006). Indeed, the E-value of these two proteins to the query was over 125 and thus they were not able to be detected by PSI-BLAST. Figure 5G shows the top 50 hits by PSI-BLAST. As shown, this query does not have similar sequences in Swiss-Prot. All the hits have almost 0 -log (E-value) scores. To conclude, in this example functionally related proteins were only retrieved by the similarity of structure but not by sequence.

#### 3.5.2 Case 2: Leucine-Rich Repeat-Containing Protein 10

This is another successful example of ContactPFP, where it predicted more accurately than the sequence-based methods. The reason for this success was different from the first case. The query is a Leucine-rich repeat-containing protein 10 of mice (UniProt ID: Q8K3W2). The function of this protein includes “actin binding” (GO: 0003779), “alpha-actinin binding” (GO: 0051393), “cardiac muscle cell development” (GO: 0055013), and the protein is localized in the “nucleus” (GO: 0005634), “cytoskeleton” (GO: 0005856), “mitochondrion” (GO: 0005739), “sarcomere” (GO: 0030017), and “myofibril” (GO: 0030016). As shown in Table 4, ContactPFP predicted all GO term correctly with the highest confidence score, 1.0. For this protein, ContactPFP showed a high prediction accuracy, a Fmax score of 1.000, while it was 0.131, 0.115, and 0.160 by PFP, ESG, and Phylo-PFP, respectively.

**TABLE 4:** Predicted GO terms for Leucine-rich repeat-containing protein 10 (UniProt ID: Q8K3W2).

		Correct GO terms	Confidence Score			
			ContactPFP	Phylo-PFP	ESG	PFP
MF	GO:0003779	actin binding	1.000	0.582	0.129	0.070
MF	GO:0051393	alpha-actinin binding	1.000	0.521	0.129	0.010
BP	GO:0055013	cardiac muscle cell development	1.000	0.521	0.129	0.020
CC	GO:0005634	nucleus	1.000	0.315	0.257	0.180
CC	GO:0005856	cytoskeleton	1.000	0.201	0.129	0.050
CC	GO:0005739	mitochondrion	1.000	0.210	0.129	0.040
CC	GO:0030017	sarcomere	1.000	0.178	0.129	0.010
CC	GO:0030016	myofibril	1.000	0.155	-	0.010
		**Incorrect GO terms**	**ContactPFP**	**Phylo-PFP**	**ESG**	**PFP**
MF	GO:0005524	ATP binding	-	1.000	-	1.000
BP	GO:0006952	defense response	-	0.478	0.127	1.000
CC	GO:0030054	cell junction	-	0.360	0.861	0.130

See the caption in Table 3.

**FIGURE 6:**
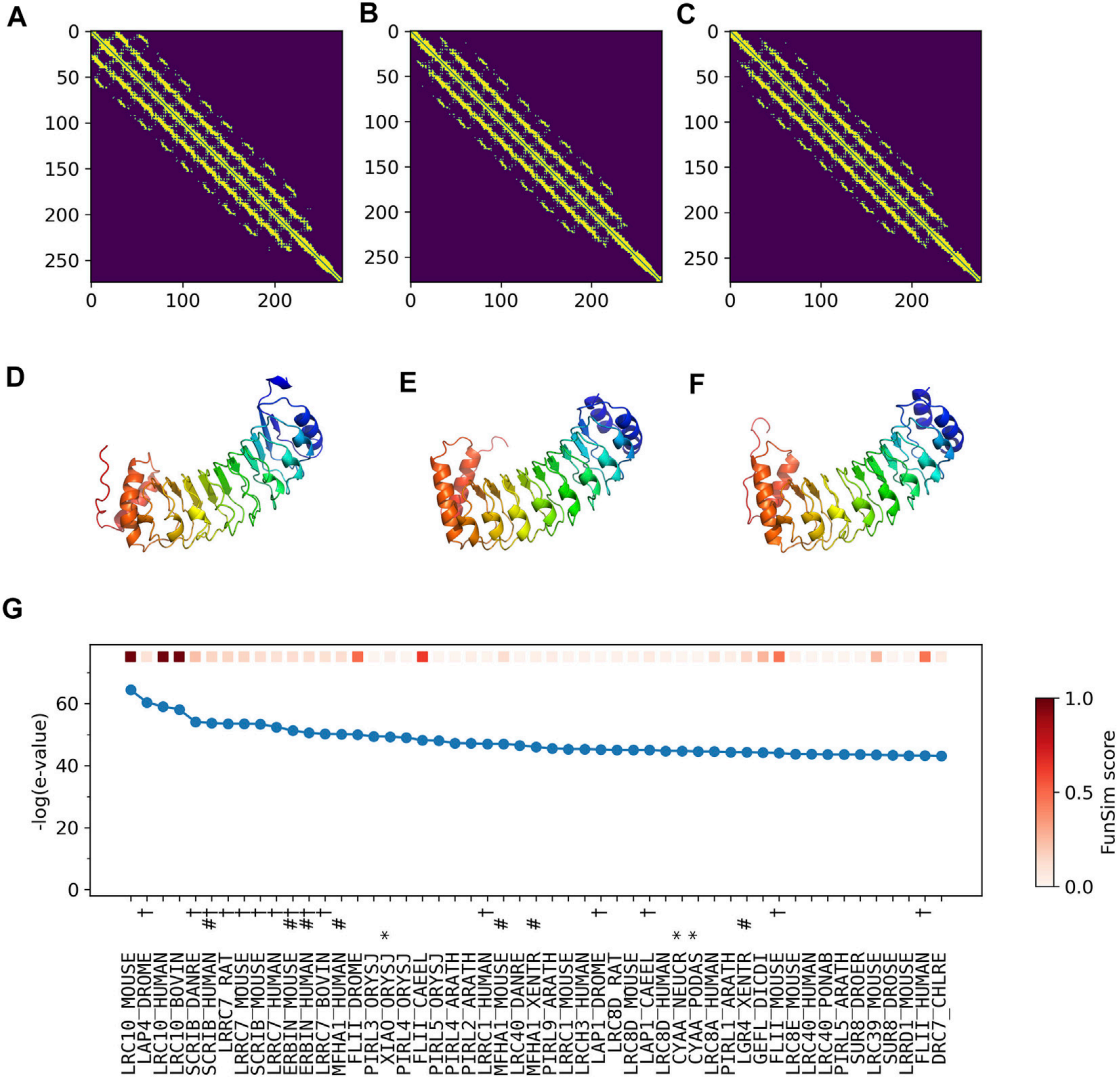
Illustration of GO term predictions by ContactPFP for Leucine-rich repeat-containing protein 10 from mouse (LRC10_MOUSE, Q8K3W2). Residue pair contact prediction of **(A)** The query, LRC10_MOUSE; **(B)** Leucine-rich repeat-containing protein from bovine, LRC10_BOVIN (Q24K06), and **(C)** Leucine-rich repeat-containing protein from human LRC10_HUMAN (Q5BKY1). The following three panels, **(D–F)**, are the corresponding predicted structures of these three proteins, respectively. The color shows the orientation of the proteins from the N-terminus to the C-terminus from blue to red. There are no experimentally determined structures for these proteins. **(G)** The top 50 hits for the query by PSI-BLAST against Swiss-Prot. Funsim scores compared with the query protein are shown in the top row in a color scale. The leftmost column is the query itself. The protein names associated with the “incorrect GO terms” listed in Table 4 are marked with the corresponding symbols, *, “ATP binding” (GO:0005524); #, “defense response” (GO: 0006952); and † “cell junction” (GO:0030054).

The correct GO term predictions by ContactPFP were transferred from the two most structurally similar proteins shown in Figure 6B,C,E,F. The query and these two proteins have a horse-shoe fold, a typical fold for proteins with Leucine-rich repeats. These two structures have high graph similarity scores of 0.917 (LRC10_BOVIN) and 0.914 (LRC10_HUMAN), respectively. These two proteins also have significant sequence similarities with E-value of 8e-59 and 1e-59, with the third and the second hits as shown in Figure 6G. However, the poor prediction accuracy by the sequence-based methods occurred because there are many other proteins with significant sequence similarity, which do not have common GO terms with the query. As shown in Figure 6G, all top 50 hits have an E-value of 10^–40^ or smaller, but only a few of them have correct GO terms. As a result, incorrect GO terms (shown in symbols) that frequently appear in the top 50 hits accumulated higher scores. Thus, in this case, the structure information was able to select the most functionally relevant proteins among proteins that are similar in sequence but not in function.

#### 3.5.3 Case 3: Cyclin-dependent Kinase Inhibitor 4

The last one is the opposite case where ContactPFP did not perform as well as the other sequence-based methods. The query is Cyclin-dependent kinase inhibitor 4 in Arabidopsis (KRP4_ARATH, Q8GYJ3). This protein has GO annotations of “cyclin-dependent protein serine/threonine kinase inhibitor activity” (GO: 0004861), “negative regulation of cell cycle” (GO: 0045786), “negative regulation of cyclin-dependent protein serine/threonine kinase activity” (GO: 0045736), “nucleoplasm” (GO: 0005654), “nucleus” (GO: 0005634), and “cytoplasm” (GO: 0005737) (Table 5). As shown in Table 5, ContactPFP predicted only one term among the correct terms and instead predicted wrong terms, including actin filament binding and organization (GO:0051015, GO:0007015), and microtubule (GO:0005874), which was worse than the other three sequence-based methods. The Fmax score of ContactPFP was 0.127, while Phylo-PFP, ESG, and PFP had a high Fmax score of 0.995.

**TABLE 5:** The detail of predicted GO terms for Cyclin-dependent kinase inhibitor 4 (UniProt ID: Q8GYJ3).

		Correct GO terms	Confidence Score			
			ContactPFP	Phylo-PFP	ESG	PFP
MF	GO:0004861	cyclin-dependent protein serine/threonine kinase inhibitor activity	-	1.000	0.923	1.000
BP	GO:0007049	cell cycle	-	0.141	0.923	0.100
BP	GO:0045786	negative regulation of cell cycle	-	0.274	0.923	0.300
BP	GO:0045736	negative regulation of cyclin-dependent protein serine/threonine kinase activity	-	0.292	0.346	0.410
CC	GO:0005654	nucleoplasm	-	0.486	0.553	0.640
CC	GO:0005634	nucleus	-	1.000	0.617	1.000
CC	GO:0005737	cytoplasm	0.988	0.053	0.005	0.180
		**Incorrect GO terms**	**ContactPFP**	**Phylo-PFP**	**ESG**	**PFP**
MF	GO:0051015	actin filament binding	1.000	0.004	-	0.010
BP	GO:0007015	actin filament organization	1.000	0.001	-	-
CC	GO:0005874	microtubule	0.988	0.002	-	0.010

See the caption in Table 3.

**FIGURE 7:**
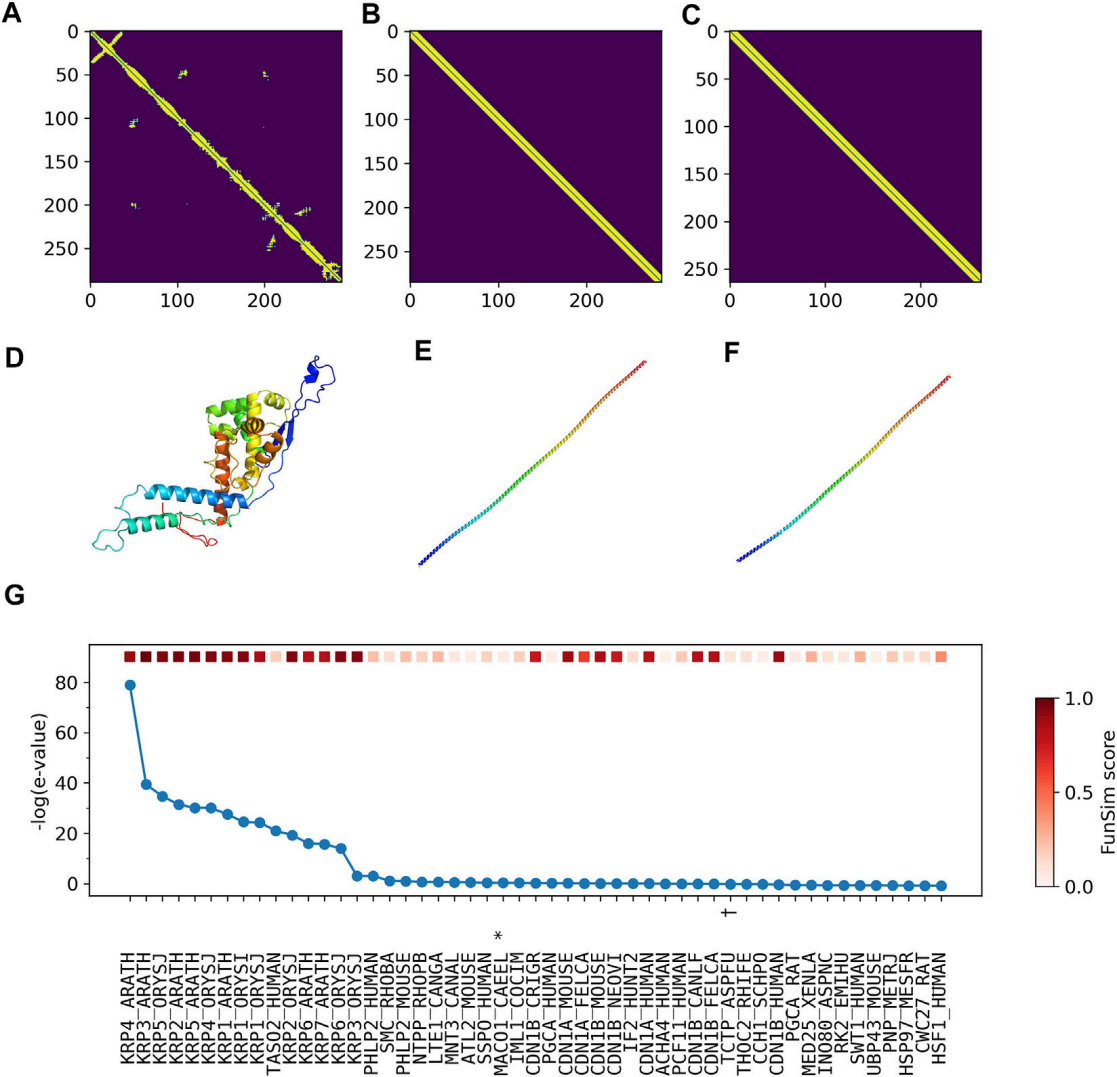
Illustration of GO term predictions by ContactPFP for Cyclin-dependent kinase inhibitor 4 (KRP4_ARATH, Q8GYJ3). The first three panels are predicted contacts for the query, KRP4_ARATH **(A)**, and the two most similar proteins in terms of the contact pattern, **(B)** TPM3_HUMAN and **(C)** TPM_CHAFE. The graph similarity scores by GR-align were 0.677 and 0.673, respectively. Panel D, E, F are predicted structures of these three proteins by trRosetta in the same order as the first row. **(G)**, The top 50 hits for the query by PSI-BLAST against Swiss-Prot. Funsim scores compared with the query protein are shown in the top row in a color scale. The most left column is the query itself. The proteins that have incorrect GO terms listed in Table 5 are marked with symbols, *, “actin filament binding” (GO: 0051015); #, “actin filament organization” (GO: 0007015); and †, “microtubule” (GO: 0005874).

According to UniProt, more than half of residues are annotated as disordered. Therefore, it is highly likely that predicted contacts (Figure 7A) and structures (Figure 7D) are incorrect. Furthermore, the top two structures selected by ContactPFP have a long, straight helical structure, which is not similar overall to the predicted structure of the query. Indeed, these two retrieved proteins, TPM3_HUMAN (Figures 7B,E) and TPM_CHAFE (Figures 7C,F) do not have any common GO terms with the query protein. In contrast, about a dozen top hits by sequence similarity search are functionally highly similar to the query, which is reflected in the high Fmax scores of Phylo-PFP, ESG, and PFP (Figure 7G). Thus, to conclude, this is an example where incorrect protein structure prediction led to the failure of ContactPFP’s function prediction.

### 3.6 Ensemble Methods

ContactPFP’s characteristic prediction performance discussed in Figure 3 motivated us to develop ensemble methods. Particularly, the primary focus is to improve predictions for proteins where ContactPFP did not perform well but other conventional methods achieved higher Fmax scores. Combining ContactPFP with other methods also makes sense from a biological point of view because the former uses protein structure information, which is complementary with the latter that uses sequence information.

**FIGURE 8:**
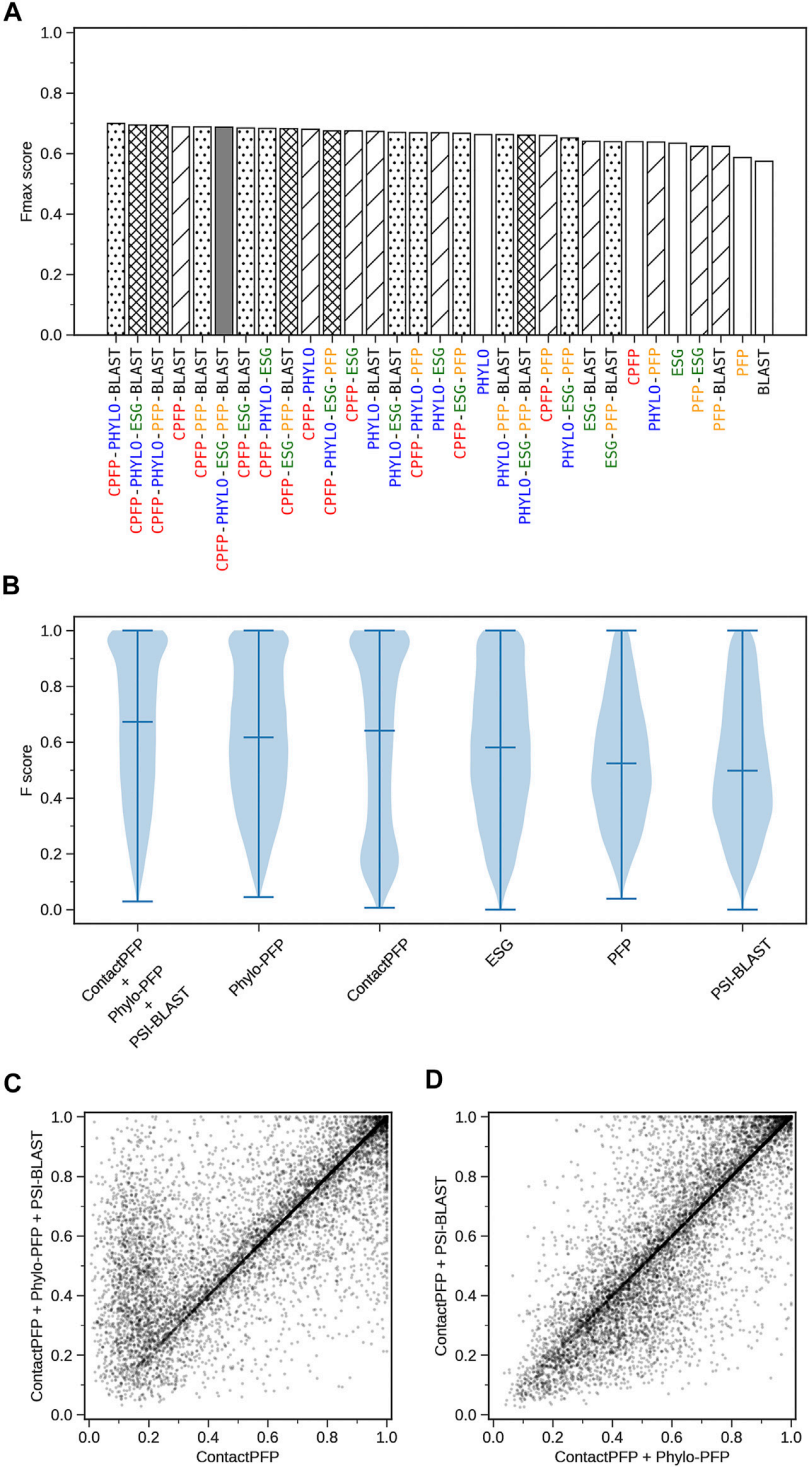
The prediction performance of ensemble methods with ContactPFP. **(A)** The average Fmax score of ensemble methods with ContactPFP. Results of all the combinations of 1–5 methods are shown. Patterns in the bar graphs show the number of methods combined. The bars are sorted by their Fmax scores. CPFP, ContactPFP; PHYLO, Phylo-PFP; BLAST, PSI-BLAST. **(B)** Fmax score distribution of the ensemble method with ContactPFP, Phylo-PFP, and PSI-BLAST, the combination with the highest Fmax score, and distributions of individual methods shown in violin plots. The three horizontal bars in a plot indicate the maximum, median, and minimum values. **(C)** Comparison of Fmax scores of individual target proteins by the best ensemble method and Phylo-PFP. Each point represents a target protein in the benchmark dataset. **(D)** Comparison of Fmax scores of individual target proteins by the ContactPFP + PhyloPFP and ContactPFP + PSI-BLAST.

We constructed ensemble methods of all possible combinations of the methods starting from single methods to a combination of all five methods (Figure 8A; Table 6). When multiple methods are combined, scores of GO terms from the combined methods were simply averaged. The highest average Fmax score, 0.699, was achieved by a combination of three methods, ContactPFP, Phylo-PFP, and PSI-BLAST. Compared with the Fmax of the lone ContactPFP, 0.638, it is a 9.6% improvement. From Figure 4A, we can see that the top methods all include ContactPFP as its ensemble component, which implies it is complementary to the other methods.

**TABLE 6:** The function prediction performance of ensemble methods.

# of methods	Ensemble name	Fmax			
		ALL	CC	MF	BP
3	CPFP-PHYLO-BLAST	0.699	0.778	0.799	0.679
4	CPFP-PHYLO-ESG-BLAST	0.694	0.774	0.795	0.672
4	CPFP-PHYLO-PFP-BLAST	0.693	0.774	0.786	0.673
2	CPFP-BLAST	0.688	0.758	0.789	0.671
3	CPFP-PFP-BLAST	0.688	0.77	0.788	0.67
5	CPFP-PHYLO-ESG-PFP-BLAST	0.687	0.771	0.784	0.666
3	CPFP-ESG-BLAST	0.684	0.765	0.795	0.665
3	CPFP-PHYLO-ESG	0.683	0.758	0.782	0.661
4	CPFP-ESG-PFP-BLAST	0.682	0.767	0.784	0.662
2	CPFP-PHYLO	0.68	0.749	0.775	0.657
4	CPFP-PHYLO-ESG-PFP	0.675	0.762	0.774	0.652
2	CPFP-ESG	0.674	0.742	0.779	0.647
2	PHYLO-BLAST	0.673	0.758	0.779	0.651
3	PHYLO-ESG-BLAST	0.669	0.753	0.777	0.646
3	CPFP-PHYLO-PFP	0.668	0.751	0.762	0.646
2	PHYLO-ESG	0.668	0.753	0.768	0.644
3	CPFP-ESG-PFP	0.666	0.748	0.764	0.644
1	PHYLO	0.662	0.75	0.759	0.641
3	PHYLO-PFP-BLAST	0.662	0.749	0.762	0.641
4	PHYLO-ESG-PFP-BLAST	0.66	0.75	0.765	0.638
2	CPFP-PFP	0.659	0.739	0.752	0.637
3	PHYLO-ESG-PFP	0.651	0.745	0.747	0.626
2	ESG-BLAST	0.64	0.728	0.76	0.612
3	ESG-PFP-BLAST	0.639	0.732	0.753	0.617
1	CPFP	0.638	0.718	0.728	0.606
2	PHYLO-PFP	0.637	0.736	0.732	0.614
1	ESG	0.634	0.714	0.746	0.598
2	PFP-ESG	0.623	0.728	0.728	0.597
2	PFP-BLAST	0.623	0.728	0.728	0.597
1	PFP	0.586	0.698	0.689	0.562
1	BLAST	0.574	0.655	0.678	0.544

All possible combinations are listed. In the column of ensemble name, CPFP, PHYLO, and BLAST correspond to ContactPFP, Phylo-PFP, and PSI-BLAST, respectively. CC, MF, BP corresponds to Cellular component, Molecular Function, and Biological Process, respectively.

Figure 8B shows the Fmax score distribution of the best ensemble method and the five individual methods. ContactPFP’s distribution has a characteristic peak at a low Fmax score of around 0.1. This peak disappeared when combined with other methods, which is the main reason why the performance improved by the ensemble method. In the score comparison of individual proteins (Figure 8C), we can also see that many proteins with a low score around 0.1 by ContactPFP improved by the ensemble method.

One thing which drew our attention is that the top combination included PSI-BLAST, which performed the worst among the five non-ensembled methods. To examine why adding PSI-BLAST improved the performance, we compared the ensemble of ContactPFP with Phylo-PFP with the ensemble of ContactPFP with PSI-BLAST (Figure 8D). We compared PSI-BLAST with Phylo-PFP because the latter was the best single method among the methods we compared (Figure 3A). From this plot, we selected two target proteins where ContactPFP + PSI-BLAST performed significantly better than ContactPFP + Phylo-PFP. APOE_HUMAN, Apolipoprotein E of Human, is one such example. The Fmax score of ContactPFP + Phylo-PFP was 0.234, while that of ContactPFP + PSI-BLAST was 0.801. This protein has 164 GO annotations. By PSI-BLAST, most of the GO terms were found at least once in the top 10 sequences thus all the GO terms had a score of 0.931 (because this is how GO terms are scored in PSI-BLAST). These GO terms were found also in the sequences retrieved by Phylo-PFP and ContactPFP. However, the GO terms were found infrequently in the retrieved sequences and thus received a low score. The same mechanism was observed for DNJA3_HUMAN, DnaJ homolog subfamily A member 3, which had an Fmax score of 0.213 by ContactPFP + Phylo-PFP and 0.988 by ContactPFP + PSI-BLAST.

### 3.7 Computational Time

**FIGURE 9:**
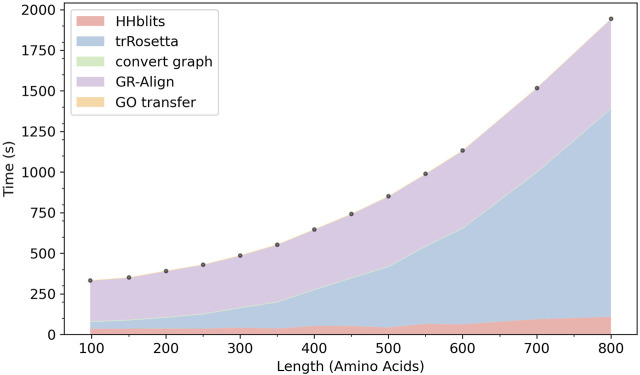
The cumulative computational time of ContactPFP. The time is decomposed into five steps: The database search with HHblits, the distance map prediction by trRosetta, converting a predicted distance map to a contact graph, contact graph comparison against the reference database by GR-Align, constructing GO term list from a hit list, are reported. The times are reported in the wall-clock time (seconds). All computations were performed on CPU, 2 AMD EPYC 7252 cores (16 cores in total) with 128GB RAM. The following 13 proteins were used, which have a length between 100 and 800 amino acids. The length of each protein is shown in the parenthesis. P0CM71 (98), P9WF14 (150), P69162 (200), B1W5S5 (250), A1YG61 (300), Q6Q972 (350), Q550G0 (400), C5A1K9 (450), Q00456 (500), A1DHW5 (550), A5DX93 (600), Q96QV1 (700), and Q54WZ0 (800). These proteins were chosen because they hit the same number of sequences, 500 (± 10) sequences, by HHblits.

In Figure 9 we show the computation time of ContactPFP. In our computational environment, the entire ContactPFP pipeline took approximately 15 min for a 500 residue-long protein. Figure 8 also shows the breakdown of the time needed for five steps in ContactPFP. The computational time for running trRosetta and reference database search by GR-Align grows as the protein length increases. Particularly, the computational time for using trRosetta grows sharply, and it exceeds the time for the reference database search when the query protein is longer than 500 residues.

## 4 Discussion

ContactPFP developed in this work identifies proteins with similar contact maps and transfers their functions to the query. Despite the knowledge that protein structure and function are closely related, protein structure information has not been effectively used for automatic protein function prediction mainly because of the low coverage of experimentally determined structure information for proteins. It is now possible to employ structure prediction methods to cover structure information of remaining proteins that have no experimentally determined structures. ContactPFP showed a slightly lower average Fmax score than one of the best sequence-based methods, Phylo-PFP, but had more wins over Phylo-PFP when predictions for individual proteins were counted. Thus, overall, we could say ContactPFP performed on par with Phylo-PFP. Combining ContactPFP in ensemble methods with sequence-based methods successfully achieved higher accuracy than the individual methods. In the current work we used simple averaging to ensemble scores from different methods. It would be worthwhile to explore other approaches beyond averaging, such as learning-to-rank, or even other signals that might indicate that a given query protein will benefit more from sequence similarity instead of structural similarity.

Since ContactPFP is based on database search, it can predict any GO terms, including very rare ones, as long as proteins found by a search have such GO annotations. This is very important for practical use of a function prediction method and is different from recent machine learning-based methods (Kulmanov and Hoehndorf, 2020; Wan and Jones, 2020; You et al., 2021), which need training on a dataset of proteins with a limited set of abundant GO terms.

Besides proving practical usefulness, we have a couple of important findings. Through comparison with sequence-based methods, we observed the strengths and the weakness of ContactPFP, which would apply to any function prediction methods that use predicted protein structures. The notable strength is that, as illustrated in the first case study, ContactPFP can often identify distantly related proteins by considering structural similarity, which leads to more accurate function prediction than sequence-based methods. ContactPFP was also able to select the most relevant proteins among proteins that are similar in the sequence (the second case study). On the other hand, weaknesses originate from the accuracy and current challenges of protein structure prediction. Structure-based retrieval does not work well when predicted structures (predicted contact maps) are not accurate. Also, handling intrinsically disordered proteins is a challenge because all disordered proteins look alike, and it is hard to distinguish functionally similar ones by their structures. An interesting finding is that the structure-based approach showed complementary strengths from sequence-based methods (Figure 3), and thus it is effective to construct an ensemble approach with other methods.

We compared simplified protein structure representations, residue contact maps as graphs, instead of directly using the three-dimensional structures. The comparison of contact maps made it possible to scan the reference database within a realistic amount of time, although it still took about 20 min for prediction on one query protein. A further speed up will be possible by using a different, efficient structure representation, such as the 3D Zernike descriptor (Venkatraman et al., 2009; Kihara et al., 2011) as it was successfully applied for real-time protein structure database search (Sael et al., 2008; La et al., 2009; Esquivel-Rodríguez et al., 2015; Han et al., 2017; Aderinwale et al., 2022).

The development of various bioinformatics tools using predicted protein structures will progress further in the future as a more recent method, Alphafold2 (Jumper et al., 2021) made significant improvements in the modeling accuracy. Function prediction (Sael et al., 2012) from predicted structures will be one such major application (Gligorijević et al., 2021). Here, we showed an approach using global protein structure comparison, but with structures, we can also identify local functional sites of proteins (Chikhi et al., 2010; Zhu et al., 2015; Sit et al., 2019) and predict binding ligands (Shin et al., 2016; Zhu et al., 2016).

## Data Availability

Publicly available datasets were analyzed in this study. This data can be found here: https://doi.org/10.5281/zenodo.6525075, https://github.com/kiharalab/contactpfp.
